# Annotations

**Published:** 1909-10-23

**Authors:** 


### Annotations.

### The Dry Shampoo.

The action of the Crown in withdrawing the charge of manslaughter against the manager and assistant of the hair-dressing department at Harrod's Stores, will meet with the approval of every medical man who has followed this interesting case with attention. After all, the action has not been entirely abortive. It has directed the attention of that large section of the community that makes use of the luxury known as a dry shampoo to the dangers which attend the use of that utterly inefficient and highly poisonous solvent, carbon tetra-chloride. On the whole, hair-dressers are much too fond of using chemicals and drugs in their business, and it may be well if some check is placed upon this tendency of theirs. Many of these most vaunted and most fashionable washes are absurd mixtures, which are impotent to effect a third of what they are supposed to accomplish. Petrol and other grease solvents are hardly justifiable when a good cake of soap, clean water, and a simple lotion are all that are necessary for an efficient and refreshing shampoo.

### The Gresham Lectures.

At the request of the Committee the lectures for the Michaelmas term will be delivered at the City of London School, Victoria Embankment, E.C. (three minutes' walk from Blackfriars Station), and not as heretofore at the Old Gresham Institute. Professor Sandwith will devote his four lectures to "Some Medical Aspects of the Poor Law Commission," and will go very fully into the important question of Poor Law reform. Thus in the first lecture he will deal with the history of the Poor Law, and will pay attention to the present relief systems, comparing and contrasting them with the methods of the past, and with the proposed scheme for the future. In the succeeding lectures he will deal with the causes, results, and treatment of pauperism in its various forms. We have always held that the Gresham lectures serve a useful and instructive purpose, and Dr. Sandwith's proposed course upholds the best traditions of the old institute. Such lectures convey a large amount of information to the public upon matters which urgently demand public consideration. One of these questions is Poor Law Reform, and it is satisfactory to note that it is at last to be popularly dealt with by the Gresham lecturer.

### Beri-beri.

A very striking paper on uncured rice as a cause of Beri-beri appears in a recent issue of the *British Medical Journal*, the author being Dr. Gilmore Ellis, Medical Superintendent and Medical Officer, Beri-beri Hospital, Singapore. It confirms very strongly the views of Dr. Braddon recently mentioned in The Hospital. The idea of rice being a cause of beri-beri is a very old one, the credit of re-establishing the theory and bringing forward definite evidence that only one kind of rice (uncured rice) is harmful belonging to Dr. Braddon, State Surgeon of Negri Sembilan, Federated Malay States. Since the Singapore Lunatic Asylum was opened in 1887, beri-beri has always been endemic there, the percentage of cases rising to such an extent that in 1896, out of an average daily number of 233 patients resident in the asylum, 165 were treated for beri-beri and 40 died. Such a place was, therefore, very suitable for testing the hypothesis of cured *versus* uncured rice, and this was done. In 1904 all patients were kept on cured rice till October the 16th without a single case of beri-beri appearing, whereas a change to uncured rice at that date produced 15 cases in December with one death. In 1906 the first 11 months of the year were run on cured rice with no cases at all, this being followed in 1907, where the patients were alternately fed for four months at a time on cured and uncured rice respectively, by a serious outbreak. These figures so convinced Dr. Ellis of the value of cured rice that since that time it has been exclusively used with the splendid result of no recurrence of the disease for over a year. That one of the causes of beri-beri has been discovered, this experiment, with others also recently carried out, pretty clearly proves. All Government institutions should certainly never include any form of uncured rice in their dietaries in the future, and though we do not yet know in what way uncured rice is dangerous, nevertheless this is only of secondary interest as compared with the fact that its disuse causes the disappearance of beri-beri.

### The Late Professor Lombroso.

To be the subject of extreme estimates, both adverse and favourable, is the lot which usually distinguishes a radical innovator. To some (especially British) anthropologists and psychiatrists Lombroso was a crank, and something of a charlatan. To others, and among them one of Nordau's calibre, he appears as "one of the loftiest mental phenomena of the nineteenth century." And yet there is not a great deal of originality about Professor Lombroso's best known propositions; he was no intellectual revolutionist; and the estimate posterity comes to form of him may well be somewhere between the two appreciations already given—a good deal higher than the one, some way below the other. With his views on spiritualism and kindred subjects we are not here concerned; superficially, at any rate, they are not reassuring. But it needs pointing out that the conception of the "degenerate," which by literary and other research Lombroso did so much to popularise, and, on the Continent, in a measure to establish, pervades modern Latin medicine and literature to an extent which should cause in this country uneasy suspicions of British insularity. Professor Lombroso's recent visit to an educational congress here passed, for instance, quite unnoticed.

